# Neuromodulation to improve gait and balance function using a sensory neuroprosthesis in people who report insensate feet – A randomized control cross-over study

**DOI:** 10.1371/journal.pone.0216212

**Published:** 2019-04-30

**Authors:** Sara R. Koehler-McNicholas, Lori Danzl, Alana Y. Cataldo, Lars I. E. Oddsson

**Affiliations:** 1 Minneapolis Department of Veterans Affairs Health Care System, Minneapolis, MN, United States of America; 2 Division of Rehabilitation Science, Department of Rehabilitation Medicine, Medical School, University of Minnesota, Minneapolis, MN, United States of America; 3 Recanati School of Community Health, Ben Gurion University of the Negev, Be’er Sheva, Israel; 4 RxFunction Inc., Eden Prairie, MN, United States of America; University of Toronto, CANADA

## Abstract

Peripheral neuropathy may cause loss of sensory information from plantar cutaneous mechanoreceptors that is important for balance control and falls management. The current study investigated short-term effects of using Walkasins, an external lower-limb sensory neuroprosthesis, on clinical outcomes of balance and gait in persons who reported peripheral neuropathy and balance problems. The device replaces lost plantar sensation with tactile balance information that modulates cutaneous mechanoreceptors above the ankle where sensation is intact. Thirty-one male community-dwelling Veterans, 56–84 years old with insensate feet and balance problems participated. Initial Functional Gait Assessment, gait speed, and 4-Stage Balance Test outcomes were assessed. After initial assessment, subjects were randomly assigned to either wearing Walkasins turned ON, or OFF, and outcomes were re-assessed following a set of standardized balance exercises. Following a one-hour rest and washout period, treatments were crossed-over between groups and a third outcomes assessment was performed. Before cross-over, 10 of 15 subjects in the ON-then-OFF group improved their Functional Gait Assessment score by at least four points, the Minimal Clinically Important Difference, compared to 5 of 16 in the OFF-then-ON group. After cross-over, 7 of 16 subjects in the OFF-then-ON group improved by at least four points versus 2 of 15 in the ON-then-OFF group. ON treatment was associated with a Functional Gait Assessment improvement of 4.4 ± 3.7 points versus 1.5 ± 1.2 for the OFF treatment (p<0.01). Overall, Functional Gait Assessment scores changed from 15.2 ± 4.8 at initial assessment to 21.1 ± 5.2 after final assessment (p<0.001). At the end of the two treatment sessions, 16 of the 31 individuals had improved their Functional Gait Assessment score beyond 23, indicating normal fall-risk status. Future studies should investigate long-term benefits of the device to reduce fall risk and actual falls in patients with peripheral neuropathy and balance problems.

## Introduction

Peripheral neuropathy (PN) is a debilitating condition that affects an estimated 30 million Americans according to The Foundation for Peripheral Neuropathy [1]. Chronic symptoms of PN often involve pain and weakness of the lower limbs as well as loss of sensory information from plantar cutaneous mechanoreceptors that has been shown to be important for postural control and balance in a series of studies by Meyer et al. [2,3]. Furthermore, epidemiological evidence has linked PN patients to an increased risk of falling [4,5] and decreased stability while standing [6,7] and when exposed to external postural perturbations [7]. An estimated 4.8 to 6.4 million Americans with diabetes, comprising 30–40% of the U.S. diabetic population, exhibit symptomatic diabetic PN [8,9] that is associated with increased fall risk [10]. The prevalence may be as high as 50% in diabetics over 60 years of age [8,9]. In the elderly population, the prevalence of PN may be as high as 20% [11]. Also, a growing number of studies are reporting gait and balance problems and an increased risk of falls in patients suffering from chemotherapy-induced PN, commonly in women breast cancer survivors [12–15]. Lower-extremity strength training may be helpful in improving balance in patients with PN [16] and supervised balance training could help lower fall risk in older adults with type 2 diabetes [17]. Furthermore, Oddsson et al. have shown that specific balance training [18] can improve balance function in healthy elderly [19] as well as in those with a fear of falling [20]. However, long-term effects and sustained benefits of such interventions may be limited unless training is continuously maintained [21,22]. Consequently, there is a growing need for developing new interventions for improving balance and mobility and to manage fall risk in the elderly [23–25] and in other clinical populations [4,5].

A growing number of studies have found benefits of using vibrotactile sensory cues to enhance balance control (see [26] for a review). Previous work by the U.S. Navy has even shown that vibrotactile displays can be used to provide navigational cues allowing blindfolded pilots to successfully control their aircraft, dramatically illustrating how the brain can incorporate novel sensory cues into functional behavior to improve performance [27,28]. More recently, studies have demonstrated the utility of tactile sensory cues to improve postural control in patients with balance problems [29–32]. Three different concepts of utilizing tactile sensory stimulation to improve sensory function were proposed by Shull and Damian [33]. They classified usage areas of “haptic” wearables according to the degree of sensory impairment in the individuals intended to use them. First, according to their classification [33], sensory substitution involves the situation when patients have completely lost a certain sense and the technology is used to replace the lost function, therefore acting as a prosthesis for the lost sense. The sensory substitution or prosthesis concept of use of tactile feedback has been applied to improve vestibular function [34–37], vision [38] and plantar pressure sensation [37]. Sensory substitution devices to aid balance have provided auditory [39–42], electrotactile [34], or vibrotactile sensory information [35,36,43–45]. Second, the concept of sensory augmentation would apply when there is partial sensory impairment where patients would benefit from supplementary sensory cues to enhance motor control, e.g., during rehabilitation activities [33] including telerehabilitation therapy [46]. Third, when there is no impairment involved, Shull and Damian proposed the term “trainer” for non-clinical use of tactile sensory information [33].

Ma et al. [26], in their review of balance improvements related to the use of wearable sensory systems, concluded that most systems improve standing balance. However, they argued the need to further study potential benefits during dynamic balance activities including gait. They further indicated the need for randomized control trials to study the effects of these technologies [26]. In a separate study, Ma et al. [45] reported improved standing balance control in subjects using a system that provided tactile sensory information of plantar pressure under conditions of simulated reduced plantar sensation using five layers of socks, suggesting clinical benefits of sensory substitution technologies.

The Walkasins, a new sensory substitution technology, is a non-invasive neuroprosthesis intended to replace lost plantar sensation in patients with sensory PN who have balance problems. The device consists of two components: a leg unit and a foot pad (see Methods). The foot pad contains sensors that measure plantar pressure to estimate anteroposterior and mediolateral excursion of the center of pressure under the foot during standing and walking activities. The foot pad connects to the leg unit that wraps around the lower leg of the user. The leg unit contains electronics for reading foot pad pressure signals and a microprocessor running an algorithm that activates four small motors that provide gentle tactile sensory stimuli to the front, back, medial, and lateral skin of the user’s leg. These new sensory cues display real-time plantar pressure information at a location above the ankle and proximal to the PN lesion where mechanoreceptors in the skin are still intact. During standing, no stimulus is provided when the center of pressure sway is located within a safe, central area. Outside of this safe zone, a stimulus specific to the location and direction of center of pressure sway occurs to indicate a potential out-of-balance event. During walking, stimuli are provided to indicate the stance phase of the gait cycle, at heel-strike and toe-off, respectively. The leg unit has a button to activate the unit and two status LEDs. Power is supplied by a rechargeable internal battery. Using an early prototype of the same technology [37,47], pilot studies have shown improved Dynamic Gait Index (DGI) scores in a group of elderly at-risk fallers.

The goal of the current study was to investigate the short-term, within-subject effects of using Walkasins on clinical outcomes of balance and gait, specifically in persons who experience balance problems and have lost sensation in the plantar surface of the foot due to PN. Specifically, we hypothesized that subjects classified as at-risk fallers who receive a sensory neuromodulation stimulus when wearing the device turned ON would improve their Functional Gait Assessment (FGA) score by at least four points, the Minimal Clinically Important Difference (MCID) for community dwelling elderly individuals [48], whereas with the device turned OFF they would not. The FGA is a modified version of the DGI that includes higher level balance tasks and has less of a ceiling effect than the DGI [49]. Study design and early findings have been presented in abstract form [50].

## Methods

### Recruitment

Human subject testing was approved according to the Declaration of Helsinki by the Institutional Review Board (IRB) Subcommittee, the Subcommittee on Research Safety, and the Research and Development Committee of the Minneapolis VA Health Care System (MVAHCS): 4646-B The Effect of Walkasins on Balance and Gait in People with Peripheral Neuropathy (see attached Protocol). The device was considered a non-significant risk device by the MVAHCS IRB, as it does not meet the criteria of a significant risk device according to U.S. Food and Drug Administration regulations. The study was registered on ClinicalTrials.gov (#NCT02115633). At the time of this study, Walkasins were only available for research purposes as approved by an IRB.

**Fig 1:**
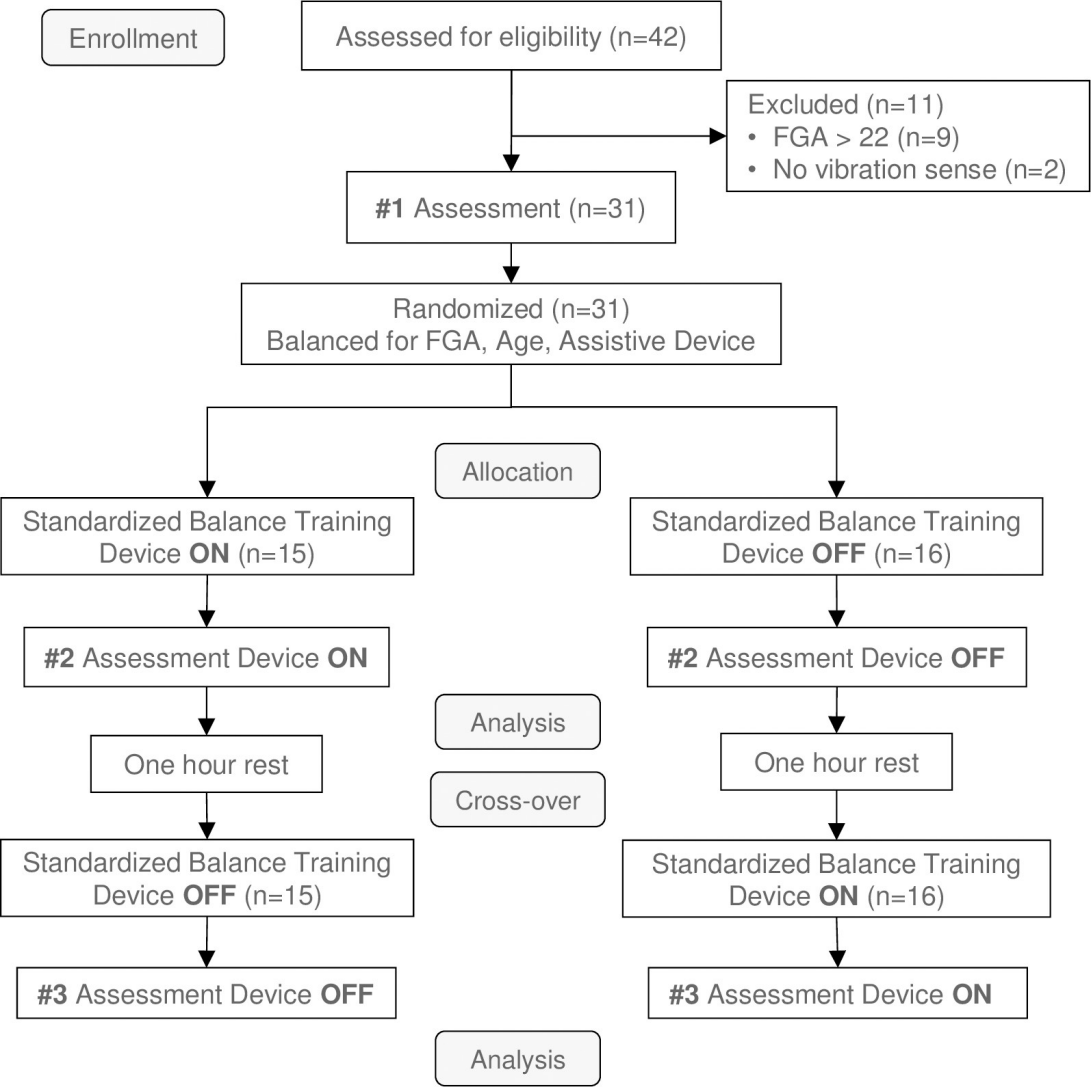
CONSORT flow chart of the study.

**Fig 2:**
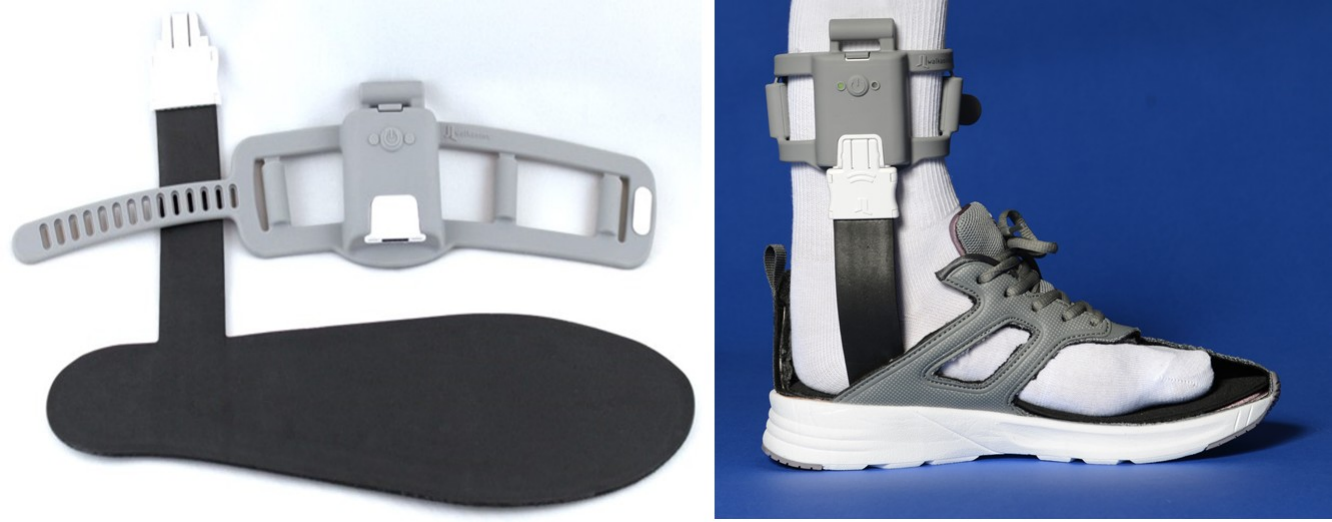
The two components of the Walkasins device, the foot pad and leg unit (left), as worn on the medial side of the leg with foot pad inserted in the shoe and leg unit wrapped around the leg above the malleolus (right).

The flow chart in Fig 1 shows an overview of the study. Data were collected on 31 Veterans who were either referred from Physical Therapy or Primary Care as candidates relevant for the study (n = 8), or potential subjects who responded to announcements posted in the hospital that specifically targeted individuals with PN and balance problems. Subjects either had a diagnosis of PN, confirmed from their medical record (n = 27), or they reported insensate feet during pre-screening conducted over the phone that was confirmed during enrollment with 10 g Semmes-Weinstein monofilament testing on the plantar surface of the foot at the big toe, 5^th^ and 1^st^ metatarsal. Inclusion criteria for the study were: age 18–90 years; male or female; insensitive feet related to sensory PN (tested with a 10 g monofilament on the plantar surface of the foot at the big toe, 5^th^ and 1^st^ metatarsal); self-reported difficulty with balance; an FGA score less than 23 (indicating high fall risk [48]); ability to understand informed consent; currently living in the community; and having a shoe/foot size between women’s 5 and men’s 12 based on specifications of the device. Subjects with larger feet were allowed into the study if they were able to activate all four motors when testing the device. Candidates reported symptoms consistent with neuropathy, including numbness, tingling, burning, and pain in addition to gait and balance problems. Subjects who were unable to perceive the stimuli from the Walkasins leg unit (Fig 2), were dependent on an ankle-foot orthosis that could not be worn with the device, had open wounds on the foot or calf, or had a musculoskeletal or neurological condition that affected gait and balance were excluded from participation. Subjects’ use of an assistive device (cane or walker) was allowed, but not during any clinical outcome assessments.

### Study procedure

The study was conducted in a hospital outpatient setting. All subjects signed a consent form approved by the MVAHCS IRB prior to the initiation of study activities. Following the informed consent process, a physical therapist (LD) performed sensation and vibration testing to document loss of sensation and to ensure subjects could feel the stimuli from the Walkasins leg unit (Fig 2).

Subjects then completed a health screening questionnaire to assess common health issues related to neurological, musculoskeletal, cardiopulmonary disorders, or other systemic diseases, as well as information on their history of falls over the past six and twelve months, regular use of an assistive device, and type of current residence (e.g., community versus nursing home). Subjects also completed the Activities-Specific Balance Confidence (ABC) Questionnaire, which asks the questions: “*How confident are you that you will not lose your balance or become unsteady*” when performing 16 different tasks [51]. The ABC was developed to measure levels of balance confidence in elderly persons. Subjects rated themselves on a scale from 0 to 100, and an average score was calculated across the 16 responses. An ABC score below 67% has been associated with high fall risk [52].

**Fig 3:**
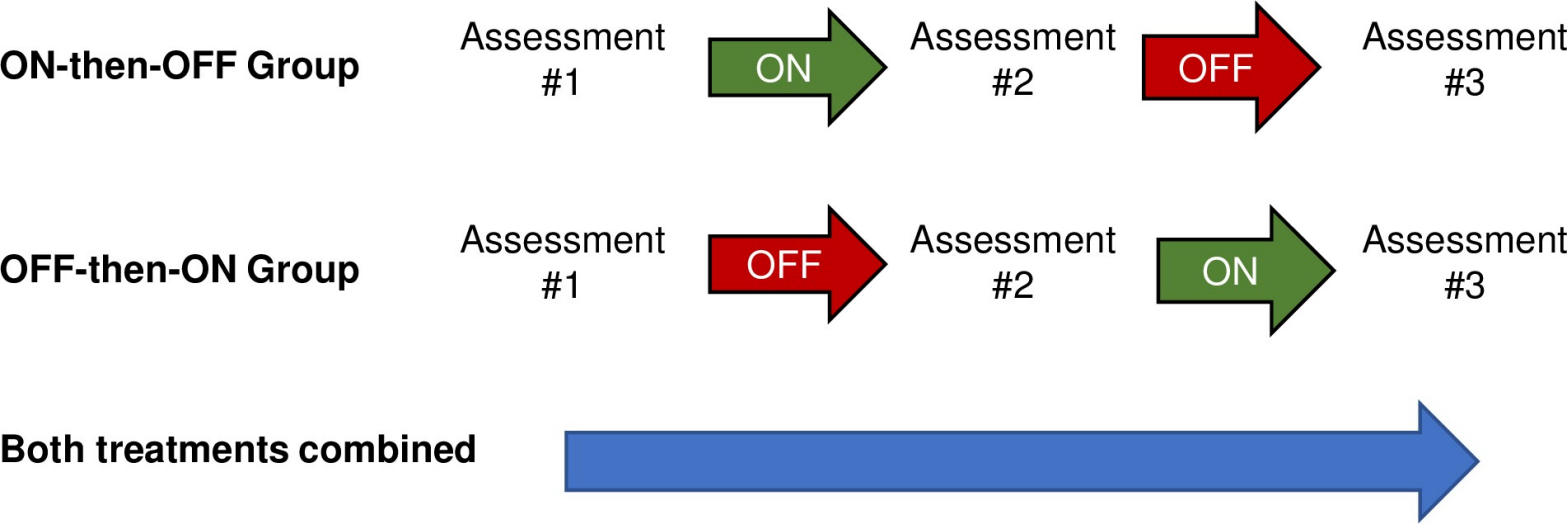
Outline of assessments and treatments for the ON-then-OFF and OFF-then-ON groups. Colors illustrate how data was pooled for analysis (see Statistical analysis).

Following completion of the questionnaires, subjects performed a series of functional outcome measures while wearing the device turned OFF (Assessment #1). The assessments were repeated two additional times during the study session (Assessment #2 and Assessment #3), each time by the same physical therapist (LD) (see Fig 3). The functional assessments are described below:

#### Functional gait assessment

The FGA is a commonly-used, reliable, and valid measure of gait function related to postural stability and has been shown to be effective in classifying fall risk in older adults and predicting unexplained falls in community-dwelling older adults [49,53]. The FGA has also been validated in stroke survivors [54] and patients with Parkinson’s disease [55]; it also has less flooring and ceiling effect than the DGI [54]. The FGA includes a 10-item scale where each item is scored from 0 to 3 (3 = normal, 2 = mild impairment, 1 = moderate impairment, 0 = severe impairment). The sum of the individual item scores represents the FGA and the maximum achievable score is 30. An increase larger than four points is considered the MCID for community-dwelling elderly individuals [48]. In the current study, subjects were considered “responders” if they improved their FGA by at least four points while participating in the study [48].

#### 10-meter walk test (10MWT)

The 10-meter walk test [56] is routinely used in rehabilitation and has been shown to have excellent reliability in chronic stroke patients [57]. Gait speed has been found to be an important predictor of survival in older adults [58], emphasizing its importance as a clinical outcome measure. Gait speed (10-meter walk using the middle 6 meters) was assessed under two conditions: 1) normal speed and 2) fast speed. A difference of 0.10 m/s of the normal speed has been defined as the MCID [56]. A change in gait speed larger than 0.13 m/s was considered “substantial meaningful” and “small meaningful” if it was larger than 0.05 m/s as proposed by Perera et al. [56].

#### 4-stage balance test

The 4-Stage Balance Test is part of the STEADI protocol recommended by the Centers for Disease Control and Prevention to assess fall risk in elderly individuals [59]. It includes four gradually more challenging postures the subject performs: 1) stand with feet side by side; 2) stand with feet in semi-tandem stance; 3) stand with feet in tandem stance; 4) stand on one leg. Subjects pass if they can hold the stance for 10 s and then move on to the next stance. A fail during tasks 1, 2 or 3, indicates a high risk of falling, i.e., a total performance time of less than 30 s. The time on each test was recorded, then summed to calculate a total score.

After subjects had completed Assessment #1 (FGA, 10MWT, and 4-Stage Balance Test), their FGA score was calculated and subjects with an FGA score <23 were randomly assigned to either perform Assessment #2 with the device turned ON (ON-then-OFF group) or OFF (OFF-then-ON group, see Fig 1 and Fig 3). For Assessment #3, treatments were crossed-over (i.e., the ON-then-OFF group was assessed with the device turned OFF and the OFF-then-ON group was assessed with the device turned ON). Subjects were randomized using minimization [60], with groups balanced according to their Assessment #1 FGA score, age, and use of an assistive device (Fig 1). The procedure for randomization by minimization outlined in [60] was implemented using an Excel spreadsheet with entries for subjects’ FGA score from Assessment #1, age, and use of assistive device. Group assignment occurred immediately after Assessment #1 and was conducted by the study coordinator. Subjects were blinded as to which group they were assigned. The cross-over design was applied to account for potential learning effects from repeating the functional outcome measures and to increase the power of the test since participants served as their own control, thereby decreasing issues with confounding factors [61,62]. The underlying condition in these patients was chronic and stable during the study period, two important conditions for the appropriate use of a cross-over design [63].

Following randomization and group assignment, subjects completed a brief standardized treatment protocol with the device in the appropriate mode (ON or OFF) according to their group assignment (ON-then-OFF group, or OFF-then-ON group, Figs 1 and 3). Consequently, both groups received the same treatment with the physical therapist (LD) although the device was either turned ON or OFF. This study design allowed the effect of the treatment protocol to be examined relative to Assessment #1, when subjects wore the device, but turned OFF. The training protocol lasted approximately 10 min and included a set of standing balance exercises (two-leg standing, tandem standing, and one-legged standing) as well as walking exercises (walking in a straight line, in circle right and left, at normal and fast speed) that were performed with the physical therapist (LD). During standing exercises subjects were challenged to explore their base of support in both mediolateral and anteroposterior directions and to notice the stimulus pattern in the case that the device was turned ON. All exercises were repeated under eyes closed conditions.

Following the first training session, subjects performed Assessment #2 (FGA, 10MWT, and 4-Stage Balance Test). The ON-then-OFF group was tested with the device turned ON and the OFF-then-ON group with it turned OFF (Fig 3). After Assessment #2 was completed, subjects were given a 60-min rest period when no testing was performed and the Walkasins were not worn. The rest also served as a washout period to decrease any carryover effects from the initial treatment. When subjects returned from the 60-min washout period, the Walkasins were donned and placed in the alternate mode (see Figs 1 and 3). The standardized treatment protocol was repeated a second time before subjects performed Assessment #3. All study activities occurred during a single study visit at the MVAHCS.

A sample size estimation was performed to detect a within-subject FGA change of at least four points (the MCID according to [48]) using a standard deviation of 5.5 (average of reported standard deviations in older adults [53,55,64]). This analysis resulted in a sample size of at least 26 subjects to achieve a 0.025 significance level and 90% power (cf. [65]). An attrition rate of 15% was applied resulting in a preferred sample size of 31.

### Statistical analysis

Descriptive statistics were calculated and presented as mean and standard deviation of the mean. Test for normality was conducted using the Shapiro-Wilk’s test. Several analyses were considered to evaluate the efficacy of the 60-min washout period administered between Assessment #2 and Assessment #3 to decrease any carryover effect (e.g., see attached Protocol). Ultimately, the potential of a carryover effect was calculated according to recommendations by Wellek and Blettner [62] by comparing the sum of the FGA scores across all three assessments between the two groups using a t-test for independent samples. Statistical significance (p<0.05) would indicate presence of a carryover effect [62]. Comparison of subject characteristics and initial outcomes between the ON-then-OFF group and the OFF-then-ON group at Assessment #1 was performed with a t-test for independent samples for interval data and the two-proportion Z-test for proportion-based measures. The following established outcomes cut-offs were used to classify participants as responders or non-responders following a treatment session: an increase in FGA score by at least four points (indicating the MCID for community-dwelling elderly individuals [48]), an increase in normal gait speed higher than 0.13 m/s (reported as “substantial meaningful” [56]), and a 4-Stage Balance Test score beyond 30 s [59].

Fig 3 illustrates how the different assessment data sets were analyzed. Effects of the OFF-treatment sessions were analyzed by pooling pre- and post-data from each subject’s OFF session (red arrows in Fig 3), i.e., the difference between Assessments #1 and #2 for the OFF-then-ON group and between Assessments #2 and #3 for the ON-then-OFF group, respectively. Correspondingly, within-subject effects of the ON-treatment sessions were analyzed by pooling pre- and post-data from each subject’s ON session (green arrows in Fig 3), i.e., the difference between Assessments #1 and #2 for the ON-then-OFF group and between Assessments #2 and #3 for the OFF-then-ON group, respectively. Within-subject effects of the combined ON and OFF treatment sessions were analyzed by comparing data from Assessment #1 to Assessment #3 across all subjects (blue arrow in Fig 3). Comparing Assessment #1 to Assessment #3 would show the effect of receiving two treatments, one with the device turned ON and one with it turned OFF. All within-subject comparisons were performed with a t-test for dependent samples. To conduct the within-subject comparisons, data sets from each of the three assessments were utilized twice (see Fig 3). To reduce risk of type I errors due to multiple t-tests, a Bonferroni correction was implemented. A p-level of less than 0.025 was considered statistically significant (correction for two t-tests on each data set, 0.05/2). The Analysis-ToolPak module in Microsoft Excel 2016 was used for statistical calculations.

Effect sizes were calculated according to recommendations by Lakens [65] using Hedges’ g_av_, an effect size measure of the d family that is commonly referred to as the corrected effect size due to less bias than Cohen’s d_av_ [65]. The 95% confidence intervals of the effect sizes were estimated according to Algina et al. [66]. Effect sizes were interpreted according to recommendations by Cohen [67], 0.2 representing a small effect, 0.5 a medium effect, and 0.8 a large effect.

## Results

Fig 1 shows the CONSORT flow diagram of the study. Forty-two subjects were enrolled and assessed for their eligibility to participate between July 15, 2016 and July 28, 2017. Eleven subjects were excluded, nine of which had an FGA score larger than 22 points (i.e., indicating normal-level fall risk) and two of which were unable to perceive the stimuli from the device (i.e., an exclusion criterion for participation in the study). One of the 42 subjects was female, although this subject did not meet inclusion criteria. Overall, 31 subjects met inclusion criteria and were included for allocation. Twenty-seven of these subjects had a diagnosis of PN in their medical record, described as diabetic neuropathy (n = 14), idiopathic/unspecified neuropathy (n = 8), or neuropathies possibly related to alcohol dependence (n = 4) or b12 vitamin deficiency (n = 1). Exposure to agent orange was noted in the medical record of eight subjects.

### Assessment #1

Table 1 shows characteristics of the 31 subjects who participated in the study after they were randomized to the ON-then-OFF group or the OFF-then-ON group. The age range of the subjects was 56–84 years. There were no statistically significant differences between the two groups with respect to outcomes or anthropometrical characteristics at Assessment #1 (Table 1). Both groups had ABC scores lower than 67%, which has been associated with an increased risk of falling [52].

**Table 1:** Characteristics of the subject population and their outcomes of Assessment #1.

Assessment #1 Randomization	ON-then-OFF group n = 15	OFF-then-ON group n = 16	p-level
Gender Male (n)	15 of 15 (100%)	16 of 16 (100%)	0.80
Gender Female (n)	0 of 15 (0%)	0 of 16 (0%)	n/a
Use of Assistive Device (n)	7 of 15 (47%)	8 of 16 (50%)	0.85
Fallen in last 6 months (n)	9 of 15 (60%)	7 of 16 (44%)	0.37
Fallen in last 12 months (n)	10 of 15 (67%)	6 of 16 (38%)	0.10
Diabetes diagnosis (n)	7 of 15 (47%)	8 of 16 (50%)	0.85
Reported difficulty walking (n)	14 of 15 (93%)	16 of 16 (100%)	0.29
Inner Ear Problems (n)	6 of 15 (40%)	4 of 16 (25%)	0.37
Hypertension (n)	11 of 15 (73%)	12 of 16 (75%)	0.91
	**Mean (SD)**	**Mean (SD)**	**p-level**
Age (yrs)	71.6 (7.1)	71.6 (6.2)	0.99
Height (m)	1.79 (0.09)	1.79 (0.08)	0.99
Weight (kg)	98.1 (15.0)	96.9 (16.7)	0.84
BMI (kg/m^2^)	30.6 (4.9)	30.2 (4.9)	0.80
FGA Score	15.0 (4.5)	15.4 (5.2)	0.80
Gait speed Normal (m/s)	0.92 (0.24)	0.94 (0.20)	0.81
Gait speed Fast (m/s)	1.30 (0.28)	1.39 (0.30)	0.41
4-Stage Balance Test (s)	21.9 (7.3)	22.6 (7.9)	0.80
4-Stage Balance Test >30s (n)	2 of 15	3 of 16	0.68
ABC score	59.6 (19.3)	64.7 (17.3)	0.45

### Consecutive changes in FGA

**Fig 4:**
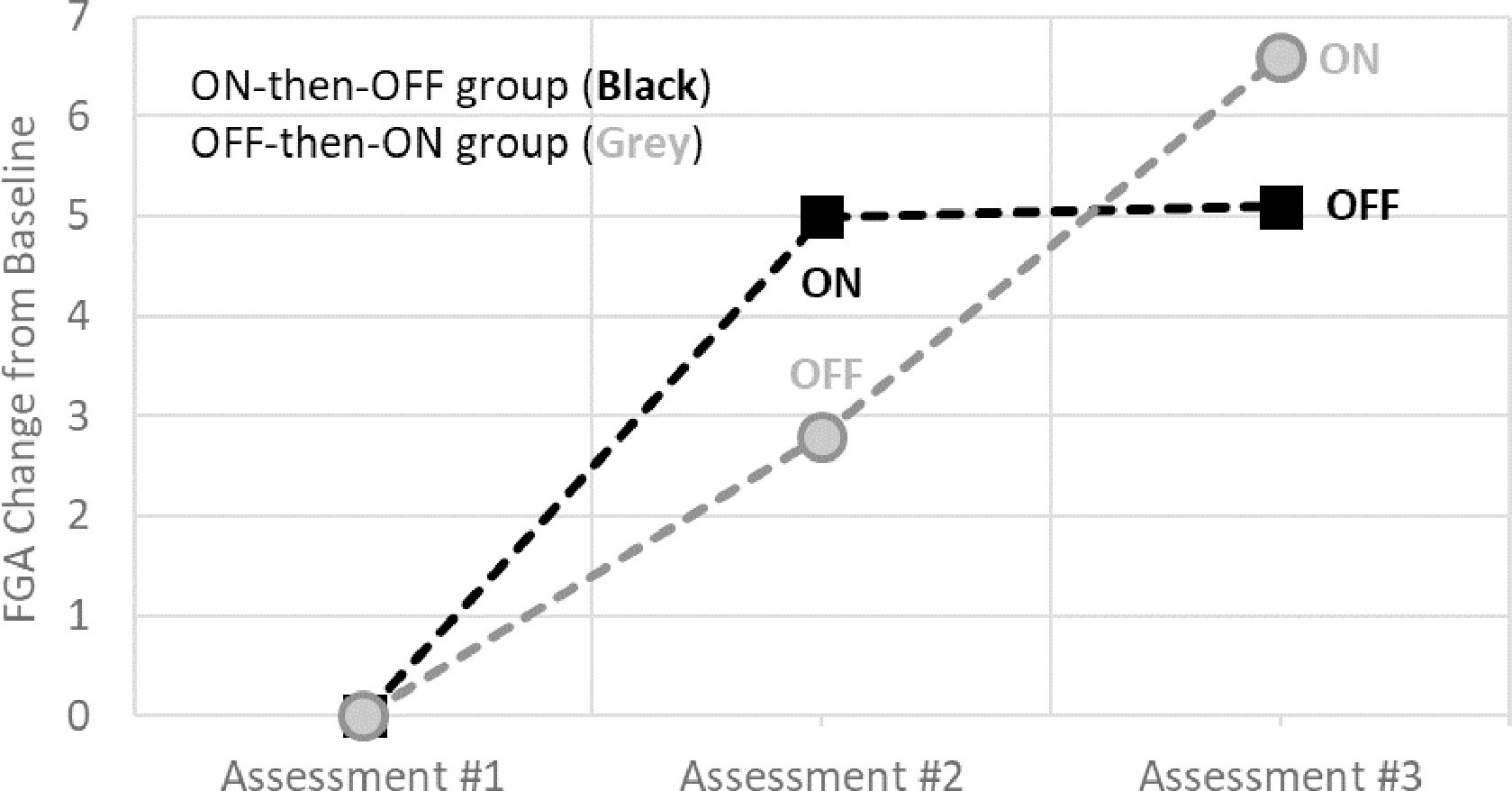
Consecutive changes in FGA scores for the ON-then-OFF and the OFF-then-ON groups.

No significant carryover effect based on sum of FGA scores across the three assessments was found (p = 0.92, [62]). Fig 4 shows consecutive changes in FGA scores across the three different assessments for each of the two groups, either using the device first ON-then-OFF or first OFF-then-ON. Larger changes in FGA score were seen when the device was turned ON, i.e., from Assessment #1 to Assessment #2 for the ON-then-OFF group and from Assessment #2 to Assessment #3 for the OFF-then-ON group. The ON-then-OFF group maintained their FGA score during their device OFF treatment at Assessment #3 (Fig 4). The OFF-then-ON group showed an improved FGA score during their device OFF session at Assessment #2.

### Proportion of subjects with FGA change of at least four points

At Assessment #2, 10 of 15 subjects (67%) in the group wearing the device turned ON improved their FGA score by at least four points compared to 5 of 16 (31%) in the group wearing the device turned OFF. The difference in proportion between the groups was statistically significant (p<0.05). At Assessment #3, 7 of 16 subjects (44%) in the group wearing the device turned ON improved their FGA score by at least four points from the prior treatment compared to 2 of 15 subjects (13%) in the group wearing the device turned OFF. The difference in proportion between the groups was not statistically significant (p = 0.063).

### Effects of ON and OFF treatment sessions

Table 2 shows pooled data from the two groups of subjects (ON-then-OFF group and OFF-then-ON group) across the two test treatments; device turned ON or turned OFF as illustrated in Fig 3. Overall, there were 24 observations of increases in FGA score of at least four points from the prior treatment across both ON and OFF treatments. These 24 observations represented 23 different subjects, i.e., one subject showed an FGA improvement of four points for both test treatments. Seventeen of the 24 observations occurred during device ON treatments and seven during device OFF treatments. Eight of the 31 subjects did not improve their FGA score by at least four points during either of the two test treatments. Consequently, 17 of the 31 subjects (55%) improved their FGA score by at least four points with the device turned ON as compared to 7 of 31 subjects (23%) when the device was OFF. The difference was statistically significant (p<0.001) and the effect size was near large (Hedges’ g_av_ = 0.79, [67]).

**Table 2:** Results comparing ON to OFF treatments.

**Effects of OFF & ON treatment sessions**	**Change after OFF treatment** **Mean (SD)**	**Change after ON treatment** **Mean (SD)**	**p-level**	**Hedges’ g**_**av**_: **(95% CI’s)**
FGA score change after OFF or ON treatment, from previous session	1.5 (3.2)	4.4 (3.7)	<0.012	0.82 (0.53, 1.14)
Number of subjects with FGA score change ≥4 (n)	7 of 31	17 of 31	<0.001	0.79 (0.18, 1.39)
**OFF & ON treatment sessions** **(Pre- and Post-values)**	**Mean (SD)** **Pre**	**Mean (SD)** **Post**	**p-level**	**Hedges’ g**_**av**_: **(95% CI’s)**
ON treatment: FGA score	16.6 (5.5)	21.0 (4.9)	<0.001	0.82 (0.51, 1.17)
ON treatment: Gait speed Normal (m/s)	0.97 (0.23)	1.06 (0.23)	<0.005	0.38 (0.15, 0.62)
ON treatment: Gait speed Fast (m/s)	1.37 (0.33)	1.43 (0.34)	<0.025	0.17 (0.01, 0.34)
ON treatment: 4-Stage Balance Test (s)	23.3 (7.9)	27.5 (7.4)	<0.017	0.53 (0.10, 0.98)
OFF treatment: FGA score	17.6 (5.5)	19.1 (5.8)	<0.015	0.24 (0.03, 0.45)
OFF treatment: Gait speed Normal (m/s)	1.0 (0.22)	1.0 (0.19)	0.67	n/a
OFF treatment: Gait speed Fast (m/s)	1.39 (0.32)	1.39 (0.32)	0.80	n/a
OFF treatment: 4-Stage Balance Test (s)	24.3 (8.0)	25.6 (8.3)	0.38	n/a

All outcomes showed statistically significant improvements after ON treatments with large effect size for FGA scores, medium for 4-Stage Balance and small for gait speed [67]. The average change in FGA score after ON treatment was 4.4 points versus 1.5 points during the OFF treatment. The difference was statistically significant (p<0.01) and the effect size was large (Hedges’ g_av_ = 0.82). After OFF treatment, only the FGA score showed a statistically significant increase although the effect size was small (Hedges’ g_av_ = 0.24) compared to the ON treatment (Hedges’ g_av_ = 0.82). No statistically significant changes were seen for gait speed or 4-Stage balance test after OFF treatment.

### Overall effects of both treatment sessions

Table 3 shows overall effects on outcomes following both the ON and OFF treatments combined for all subjects (i.e., between Assessment #1 and Assessment #3) following exposure to two balance training sessions, one with the device turned ON and one with the device turned OFF. After Assessment #3, 16 of the 31 subjects showed an increase of at least 0.13 m/s of their normal gait speed compared to Assessment #1. Fourteen of the 31 subjects performed the 4-Stage Balance Test for longer than 30 s after Assessment #3 compared to 5 subjects at Assessment #1. The change in proportion was statistically significant (p<0.05) and the effect size was large (Hedges’ g_av_ = 0.80). The mean within-subject FGA score changed from 15.2 at Assessment #1 to 21.1 at Assessment #3. The change was statistically significant (p<0.001) and the effect size was large (Hedges’ g_av_ = 1.14).

**Table 3:** Results comparing Assessment #1 and Assessment #3 representing combined effects of both treatments.

**Effects of Both Treatments** **Assessment #1 to Assessment #3**	**Assessment #1**	**Assessment #3**	**p-level**	**Hedges’ g**_**av**_: **(95% CI’s)**
FGA score change ≥4 (n)	n/a	24 of 31	n/a	n/a
Gait speed Normal increase ≥ 0.13m/s (n)	n/a	16 of 31	n/a	n/a
4-Stage Balance Test >30s (n)	5 of 31	14 of 31	<0.05	0.80 (0.14, 1.46)
Subjects with FGA>22 (n)	0 of 31	16 of 31	n/a	n/a
	**Mean (SD)**	**Mean (SD)**	**p-level**	**Hedges’ g**_**av**_: **(95% CI’s)**
FGA scores all (n = 31)	15.2(4.8)	21.1(5.2)	<0.001	1.14: (0.79, 1.56)
Subjects with FGA score >22 post sessions (n = 16)	18.3 (2.9)	25.1 (1.8)	<0.001	2.51: (1.69, 3.92)
Subjects with FGA score <23 post sessions (n = 15)	11.9 (4.3)	16.8 (4.0)	<0.001	1.11: (0.49, 1.84)
Gait speed Normal (m/s)	0.93 (0.22)	1.02 (0.21)	<0.005	0.41: (0.14, 0.69)
Gait speed Fast (m/s)	1.35 (0.29)	1.42 (0.32)	<0.05	0.22: (0.05, 0.40)
4-Stage Balance Test (s)	22.2 (7.5)	27.6 (7.5)	<0.001	0.70: (0.37, 1.06)

Sixteen of the subjects ended the third test session with an FGA score higher than 22, i.e., in the normal fall risk range. Their mean FGA score at Assessment #1 was 18.3 compared to 25.1 at Assessment #3, respectively. The difference was statistically significant (p<0.001) and the effect size was large (Hedges’ g_av_ = 2.51). The remaining 15 subjects who were still at high fall risk (FGA<23) after Assessment #3 did increase their FGA score from an average of 11.9 to 16.8. The change was statistically significant (p<0.001) and the effect size was also large (Hedges’ g_av_ = 1.11) although less than half the magnitude of the normal fall risk group’s effect size. Mean normal gait speed increased from 0.93 m/s at Assessment #1 to 1.02 m/s at Assessment #3 (p<0.005). The effect size was medium (Hedges’ g_av_ = 0.41). Fast gait speed increased from 1.35 m/s to 1.42 m/s (p<0.05) representing a small effect size (Hedges’ g_av_ = 0.22). The 4-Stage Balance test performance increased from 22.2 s at Assessment #1 to 27.6 s at Assessment #3 (p<0.001), a medium effect size (Hedges’ g_av_ = 0.70).

## Discussion

Using a randomized cross-over design, results from this study have demonstrated short-term meaningful improvements in clinical outcomes of gait and balance function associated with use of a sensory neuroprosthesis in patients who reported PN and had self-reported balance problems severe enough to significantly increase their risk for falls (FGA<23). We did not see a carryover effect, a potential concern in cross-over study designs [61–63]. Furthermore, a period effect would have been unlikely since the treatment period was short and the underlying condition in these patients was chronic and stable [61]. Following a single treatment session, sensory balance information provided by the Walkasins device more than doubled the likelihood of improving clinical outcomes of gait and balance function. Using the device turned ON, two thirds of subjects improved their FGA scores by at least four points, the MCID for community dwelling elderly individuals [48], compared to one third of subjects wearing it turned OFF. After cross-over, again more than twice as many subjects in the Walkasins ON group improved their FGA by at least four points. Seventeen out of 24 FGA improvements of at least four points occurred during device ON treatments. At the end of the two treatment sessions, 16 of the 31 individuals had improved their FGA score beyond 23 indicating normal fall-risk status. Similar trends were observed for “substantial meaningful” improvement of normal gait speed (>0.13 m/s, [56]) although overall changes in gait speed were less (Table 3).

These results confirm and further expand previous pilot studies that found improvements in the DGI in a group of elderly at-risk fallers associated with use of an earlier prototype of the device termed the “sock” [37,47]. The DGI is similar to the FGA used in the current study providing a score of dynamic gait performance that is related to fall risk [68]. The study by Wall et al. [37] compared two balance prosthesis technologies on the same group of community dwelling older adults who reported balance problems and were at high fall risk according to their DGI score. The “sock” provided sensory information based on changes in plantar pressure as measured with a sensor-instrumented shoe insert. The second device, the “vest”, developed by Wall et al. [30,35,36,44], measured magnitude and direction of trunk tilt away from the vertical and provided directional- and magnitude-specific tactile stimuli around the trunk through the vest worn by the subject. Both technologies were intended for sensory substitution, i.e., replacing either lost plantar sensation or vestibular function, respectively [37]. This previous study demonstrated a tendency for higher improvement in patients’ DGI scores when the sensory information provided corresponded to the main sensory loss of the subject. Consequently, subjects with vestibular hypofunction improved more with the vest technology, whereas subjects with PN improved more with the technology providing sensory information of plantar pressure, supporting the sensory substitution or prosthetic function of these technologies [37]. Such sensory-specific improvements could be explained by the overall functionality of the balance control system, which relies on the integration of visual, vestibular, and somatosensory information to provide optimal balance control. If one of the main sensory channels is compromised, it seems reasonable that a prosthesis for that specific sense would provide the largest opportunity for improvement of balance function, further supporting the concept of sensory substitution [33]. Interestingly, subjects did show some improvement of their DGI score with both technologies, suggesting the potential for a sensory augmentation effect [37]. In the elderly population, there is likely a general age-related loss of balance function across all sensory channels, which could explain a sensory augmentation effect from using a balance prosthesis.

Findings in the current study of short-term effects on gait and balance function are of interest, although the Walkasins device is intended to be worn on a continuous basis as a sensory neuroprosthesis to substitute for lost plantar sensation in patients with PN who have balance problems. Studies on long-term effects of using a neuroprosthesis to improve gait and balance function are currently limited. However, in a recent case-study, Wrisley et al. [69] reported on long-term effects in a patient with insensitive feet due to diabetic PN who had used Walkasins 8–10 hours/day over a period of four months. Within one month of daily use, this patient had increased his FGA score from 13 to 28, mini-BEST score [70] from 15 to 26, normal gait speed from 0.23 m/s to 1.01 m/s, and his ABC score from 32.5% to 73.7%. All assessments were performed with the patient wearing the device. In addition, the patient subjectively reported less pain, decreased use of pain medication, as well as improved function and quality of life [69]. Although such effects are encouraging, this one patient likely reaped benefits from an increase in physical activity he was able to perform when wearing the device. Future studies in a representative cohort of patients would be needed to further confirm long-term benefits of using a neuroprosthesis to improve gait and balance function in patients with PN who have balance problems.

In a recent study, Sienko et al. [71] reviewed use of sensory augmentation for individuals with vestibular deficits and concluded it to be most useful during various standing activities, including during mild dynamic perturbations. During gait activities, however, benefits of sensory augmentation for individuals with vestibular deficits were limited with reports of slower and less coordinated gait, at least after limited training [71]. In the current study, effect sizes in outcomes after using a sensory neuroprosthesis for lost plantar sensation were larger during FGA related gait activities as compared to standing activities (Tables 2 and 3). The FGA score, which incorporates 10 different gait activities, showed a large effect size after ON treatment (Hedges’ g_av_ = 0.82), whereas the 4-Stage Balance Test, which includes standing activities, showed a medium effect size (Hedges’ g_av_ = 0.53, Table 2). Similar observations were made for effects following both treatments combined (Table 3). In addition, several patients made comments that the device felt most helpful during walking activities. Besides targeting two different sensory channels, these two technologies interface differently with the body and the central nervous system. While sensory augmentation systems for individuals with vestibular deficits typically display sensory information to the torso [30,35,36,44,71], Walkasins display information around the lower leg, in close proximity to the plantar surface of the foot, the anatomical location of the lost sensation it is intended to replace. In fact, tactile stimuli provided by the Walkasins modulate skin mechanoreceptors along the same lower extremity dermatomes as the plantar surface of the foot (mainly tibial and sural nerve branches) at a location directly proximal to the plantar afferent projections. For this reason, it may simply be more intuitive to integrate such sensory information into functional behavior, especially during gait when lower-limb afferent information is important for environmental adaptation and is expected by the central nervous system [72,73].

While the results of this study are promising, this study has some limitations. First, neither the subjects nor the physical therapist (LD) assessing the outcomes were blinded to whether the device was ON or OFF. Blinding subjects in this type of study would have been difficult since subjects were included only if they were able to feel stimulation from the device. Also, when the device is turned ON, motor vibrations are audible. Consequently, without more sophisticated methods to obscure the sound of the device, subjects would know if the device was turned ON or OFF during the different assessments. We believe concerns regarding effects of learning and/or fatigue due to multiple assessments in the study design were addressed through the minimization randomization process [60] followed by a cross-over of treatments and by providing subjects a 60-min break before Assessment #3 was performed. Furthermore, subjects were provided rest as needed throughout the test session. Involving completely blinded therapists was unfortunately beyond the scope of the current study. Instead, we decided to use one experienced physical therapist (LD) to conduct all assessments of clinical outcomes, thereby avoiding inter-rater variability. Second, a full neurological examination to diagnose neuropathy was not conducted and was beyond the scope of the current study. Instead, we relied on a combination of medical record information and monofilament screening, which is commonly used clinically. Finally, all subjects in the current study were male. This was not surprising since the Veteran population is predominantly male, especially in the older Veteran population where PN is most common. Although PN overall is prevalent in both men and women and is likely to cause similar problems with balance, interpretation of results from the current study should be limited to the elderly male population. Future studies should target a demographically diverse and representative patient population and address long-term effects of using a neuroprosthesis to improve gait and balance function as well as quality of life in individuals with PN who have balance problems.

## Conclusions

A sensory neuroprosthesis for lost plantar pressure sensation can improve outcomes of gait and balance function in patients with PN who have balance problems. A majority of patients in the current study improved their clinical outcomes of gait and balance function after a brief session with a physical therapist. These findings suggest new sensory balance cues provided to the lower limb can modulate the activity of relevant nerve afferents and become integrated into sensorimotor control of balance and gait within a single therapy session. Additional studies are required to investigate whether these effects carry over into long-term device use to further improve gait and balance function and help decrease fall risk in community dwelling individuals with PN who experience balance problems.

## Supporting information

S1 Table Minimal data underlying study results. (XLSX)

S1 File IRB-approved protocol. (DOCX)

S2 File CONSORT_2010_Checklist. (DOC)

S3 File TIDieR_Checklist. (DOCX)
