# Supplementary material for: Neuromodulation to improve gait and balance function using a sensory neuroprosthesis in people who report insensate feet – A randomized control cross-over study
Source: PLoS One. 2019 Apr 30;14(4):e0216212. doi: 10.1371/journal.pone.0216212 (PMC6490932; doi:10.1371/journal.pone.0216212)
Supplement: S1 File — (DOCX) [file pone.0216212.s002.docx]

**MINNEAPOLIS VA HEALTH CARE SERVICE**

**INSTITUTIONAL REVIEW BOARD**

**APPROVED PROTOCOL**

4646-B: The Effect of Walkasins on Balance and

Gait in People with Peripheral Neuropathy

PI: Sara Koehler-McNicholas, PhD

Version Date: March 17, 2017

**Introduction:**

Approximately 4.8 to 6.4 million Americans, comprising 30-40% of the U.S. diabetic population, exhibit symptomatic diabetic peripheral neuropathy (PN) (Harris, Eastman et al. 1993; Martyn and Hughes 1997; Apfel 1999). The prevalence may be as high as 50% in diabetics over 60 years of age. Up to 20% of the elderly population may be affected by peripheral neuropathies (Harris, Eastman et al. 1993; Richardson and Ashton-Miller 1996). Epidemiological evidence has linked PN patients to an increased risk of falling (Richardson, Ching et al. 1992; Richardson and Hurvitz 1995) and decreased stability while standing (Geurts, Mulder et al. 1992) and when exposed to external postural perturbations (Inglis, Horak et al. 1994). Consequently, there is a need for developing cost-effective interventions for improving mobility and balance to manage fall risk in the elderly (Rubenstein, Robbins et al. 1990; Studenski, Duncan et al. 1991; Province, Hadley et al. 1995) and other clinical populations (Richardson, Ching et al. 1992; Richardson and Hurvitz 1995). Walkasins™, a lower limb sensory prosthesis that replaces lost foot pressure sensation with vibrotactile feedback around the lower calf, can address this need.

A growing body of research has investigated the use of vibrotactile feedback to enhance balance control following short-term use. Vibrotactile displays have been used successfully by the U.S. Navy to provide navigational cues that allow blindfolded pilots to control their aircraft (Rupert 2000; Rupert 2000). Sensory substitution devices to aid balance have provided auditory (Chiari, Dozza et al. 2005; Dozza, Chiari et al. 2005; Dozza, Chiari et al. 2005; Dozza, Horak et al. 2007), electrotactile (Tyler, Danilov et al. 2003), and vibrotactile feedback (Wall, Weinberg et al. 2001; Wall, Merfeld et al. 2002; Wall and Weinberg 2003; Wall, Oddsson et al. 2004). More recent work has demonstrated the utility of vibrotactile feedback to improve postural control in patients (Horak, Dozza et al. 2009; Wall, Wrisley et al. 2009; Horak 2010; Wall 2010; Wall and Kentala 2010). None of these technologies, however, have been based on the measurement of foot pressure nor have they been intended to be worn on a continuous basis as a balance prosthetic device (Statler, Wrisley et al. 2007, Wall 2012). Current devices typically require a lab-engineer “on hand” to ensure functionality.

**Hypotheses and Objectives:**

**Figure 1. The Walkasins incorporate a thin shoe insert that measures changes in foot pressure during body sway. Vibrations provided to the skin on the lower calf represent new sensory information that the patient learns to use to improve balance.**


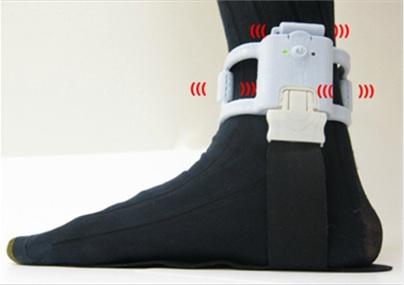


The purpose of this study is to investigate the effect of Walkasins (Figure 1), a new user-friendly and robust external lower limb sensory prosthesis developed by RxFunction Inc. under NIH funding through a Small Business Innovative Research grant, on the balance and gait of persons with peripheral neuropathy who also experience balance problems. Walkasins provide gentle vibrations to the skin around the lower leg that reflect changes in pressure under the foot sole indicating the state of balance. The user learns to use these vibrations to control balance.

We hypothesize that patients who use the Walkasins turned on will receive balance information that will improve their outcomes on gait and balance function, whereas patients who wear Walkasins turned off will not improve their outcomes on gait and balance function.

**Research Subject Recruitment and Inclusion/Exclusion Criteria:**

In this study, we will enroll up to 50 subjects to account for attrition with a final goal of 30 Veterans who have been diagnosed with peripheral neuropathy and who have self-reported balance problems. The following inclusion and exclusion criteria will be used for the study:

Inclusion criteria

- Age: 18-90 years, male or female,
- Self-reported difficulty with balance,
- Diagnosed with sensory peripheral neuropathy in one or both feet (inability to perceive 10 g monofilament on the plantar surface of the foot at the big toe, 5th and 1st metatarsal),
- Functional Gait Assessment (FGA) <23 or fail on test 1, 2 or 3 on the 4-Stage Balance Test,
- Ability to understand informed consent,
- Living in the community,
- Shoe size between 8.5’’ (women’s 5) and 11.25’’ (men’s 12).

Exclusion criteria

- Inability to perceive vibration stimulation on the lower calf (e.g., inability to sense vibration from tuning fork on the calf),
- Use of ankle-foot orthosis for ambulation,
- Open wounds on the foot or calf,
- Musculoskeletal or other neurological conditions that impact gait and balance (for example total joint replacement, moderate to severe Parkinson’s disease, stroke, etc.).

Fliers will be posted in the physical therapy gyms at the Minneapolis VA Health Care System to advertise the study. Subjects who see the flier will be asked directly about their interest in the study. Subjects will also be recruited by screening patient data within CPRS using the ICD-10 code for peripheral neuropathy. Subjects found in the screening process will first be contacted via a letter, and then called by the PI or a member of the study team to see if they are interested in participating in the study.

The study will involve one visit to the Minneapolis VA’s Physical Therapy Department and should last between 2 to 3 hours. After arriving at the VA, the subject will go through the consent process with a member of the research staff. The consent process will happen within a private room in the Physical Therapy Department. Study staff will answer any questions related to the study and will ask the subject to answer questions that verify that they understand the study. After the consent process, the subjects will sign the appropriate forms indicating their consent to participate in the study and to allow the study team to use their data (explained in consent and HIPAA documents). After the consent process, the study staff will check the inclusion and exclusion criteria and will enroll the subject into the study if they meet all criteria.

**Research Methodology:**

After enrollment into the study, subjects will complete a health screening questionnaire and the Activities-Specific Balance Confidence Scale:

- *Health Screening Questionnaire* – Subjects will fill out a screening questionnaire to assess common health issues related to neurological, musculoskeletal, cardiopulmonary disorders, and other systemic diseases as well as information on falls and currently used medications.
- *Activities-Specific Balance Confidence (ABC) Questionnaire* – Powell and Myers (1995) developed the Activities-Specific Balance Confidence (ABC) Scale in an effort to detect levels of balance confidence in elderly persons (see Appendix). The ABC scale is a one-page questionnaire that asks questions about balance confidence when performing 16 different tasks.

Subjects will then don the Walkasins device on both feet (turned off) and complete baseline measures of the Functional Gait Assessment (FGA), 10-meter Gait Speed and 4-Stage Balance Test (see Figure 2 for protocol design). Subjects may also be asked to perform the 2-Minute Walk Test. During baseline testing, the Walkasins will be turned off.

Baseline Test

30 subjects don device and are tested for FGA, Gait Speed & 4-Stage Balance with device **OFF.**

Training

15 subjects wear device and go through training with device **ON**.

Training

15 subjects wear device and go through training with device **OFF**.

Testing

15 subjects wear device and go through testing with device **ON**.

Testing

15 subjects wear device and go through training with device **OFF**.

Break

Break

Training

15 subjects wear device and go through training with device **ON**.

Testing

15 subjects wear device and go through testing with device **ON**.

Training

15 subjects wear device and go through training with device **OFF**.

Testing

15 subjects wear device and go through training with device **OFF**.

**Figure 2:** Cross-over study design.

- *Functional Gait Assessment* – The FGA is a reliable and valid measure of gait function related to postural stability and has been shown to be effective in classifying fall risk in older adults and predicting unexplained falls in community-dwelling older adults (Wrisley, Marchetti et al. 2004; Wrisley and Kumar 2010). It has also been validated in stroke survivors (Lin, Hsu et al. 2010) and patients with Parkinson’s disease (Leddy, Crowner et al. 2011) and it has less flooring and ceiling effect than the Dynamic Gait Index (Lin, Hsu et al. 2010). The FGA includes a 10-item scale where each item is scored from 0 to 3 (3=normal, 2=mild impairment, 1=moderate impairment, 0=severe impairment). The maximum score is 30. A difference of 8 points between measurement times is defined as the minimal clinically important difference (MCID) in persons with vestibular disorders (Marchetti and Whitney 2010).
- *10-meter Gait Speed* – The 10-meter walk (Perera, Mody et al. 2006) is routinely done in rehabilitation, has excellent reliability in chronic stroke patients (Hiengkaew, Jitaree et al. 2012), and physical therapists are well-trained in reliably performing this measure. In addition, gait speed has been found to be an important predictor of survival in older adults (Hardy, Perera et al. 2007), further emphasizing its importance as a clinical outcomes measure. Gait speed (10-meter walk using the middle 6 meters) will be assessed under two conditions: 1) instructed to walk at normal speed, 2) instructed to walk as fast as they can. A difference of 0.10 m/sec is defined as the MCID (Perera, Mody et al. 2006).
- *4-Stage Balance Test* – The 4-Stage Balance Test is part of the CDC recommended test protocol for balance function (STEADI, <http://www.cdc.gov/steadi/pdf/4-stage_balance_test-a.pdf>). It includes four gradually more challenging postures the subject is exposed to: 1) Stand with feet side by side; 2) Stand with feet in semi-tandem stance; 3) Stand with feet in tandem stance; 4) Stand on one leg. Subjects pass if they can hold the stance for 10 sec and then move on to the next stance. A fail of 1, 2 or 3 indicates at risk of falling. In addition, we will record time for each of the tests.
- *2-Minute Walk Test* – The 2-Minute Walk Test is an abbreviated version of the 6-Minute Walk Test that has demonstrated test-retest reliability in subjects ranging from 3-85 years old (Bohannon, Bubela, et al. 2014) and has been normed and validated as part of the Motor Battery of the NIH Toolbox (Bohannon, Wang, Gershon 2015). This test is a measurement of endurance that assesses the distance walked on a hard surface over two minutes. Subjects are instructed to walk as far as they can over two minutes and the distance is measured. This test has also demonstrated reliability among older adults (Brooks, Davis, Naglie 2007; Connelly, Thomas et al. 2009).

According to a matched-subject statistical design, subjects will then be randomized into two groups based on their gait function (as measured by the FGA) and use of an assistive device (normal use is permitted, but not during the research study). Group A will initially be trained and tested with the Walkasins turned on, then following a 60-minute break, will be trained and tested with the Walkasins turned off. Group B will initially be trained and tested with the Walkasins turned off, then following a 60-minute break, will be trained and tested with the Walkasins turned on. During testing, subjects will complete trials of Functional Gait Assessment, 10-meter Gait Speed and the 4-Stage Balance Test. All training and testing will be done by a physical therapist.

**Data Analysis:**

The primary outcome measures of this study include the Functional Gait Assessment, 10-meter Gait Speed test, and the 4-Stage Balance Test. Using a between-group parametric statistical model, we will compare baseline measures from both groups to post-training outcomes following the first training session. We expect that Group A (i.e., the group that first trained with the Walkasins on) will have a statistically significant improvement in functional gait and balance measures compared to Group B (i.e., the group that first trained with the Walkasins off). We will also compare baseline measures to the post-training outcomes for Group A following the second training session (i.e., with the Walkasins off) to confirm that the 60-minute break between training sessions allowed for a washout effect of the first training. If we are able to confirm a washout effect, we will pool our data (n=30) to compare the outcome measures from baseline and post-training with the Walkasins turned on.

**Risk to the Subjects and Approaches Taken to Minimize Risk:**

- Use of device/administration of physical stimuli - The device being tested may distract the subject while walking and increase the risk of falling. To prevent falling, a spotter will walk next to the subject to help regain balance if a fall starts to occur.
- Video recordings and photographs - We will take videos and photos of the person using the Walkasins for use in publications and presentations. There is a risk that their identity will be disclosed by being recognized in these publications and presentations. We will collect their separate consent for use of pictures and video.
- Use of medical records - We may use the medical records to obtain information related to the cause of their peripheral neuropathy, for example, to be used in describing subjects in the study. There is a risk that study personnel may inadvertently see other information in their medical record unrelated to their neuropathy that may be private in nature. We will obtain HIPAA authorization for use of the medical records.

**References:**

Apfel, S.C. (1999). Diabetic polyneuropathy CME. Diabetes and Endocrinology Clinical Management, from http://www.medscape.com/Medscape/endocrinology/ClinicalMgmt/CM.v01/pnt-CM.v01.html.

Bohannon, R.W., Bubela, D., Magasi, S., McCreath, H., Wang, Y.-C., Reuben, D., Rymer, W.Z., Gershon, R. (2014). "Comparison of walking performance over the first 2 minutes and the full 6 minutes of the Six-Minute Walk Test." BMC Research Notes **7**, 269. <http://doi.org/10.1186/1756-0500-7-269>

Bohannon, R.W., Wang, Y.-C., & Gershon, R.C. (2015). "Two-Minute walk test performance by adults 18 to 85 years: Normative values, reliability, and responsiveness." Archives of Physical Medicine and Rehabilitation **96**(3), 472-477. <http://dx.doi.org/10.1016/j.apmr.2014.10.006>

Brooks, D., Davis, A., & Naglie, G. (2007). "The feasibility of six-minute and two-minute walk tests in in-patient geriatric rehabilitation." Canadian Journal on Aging / La Revue Canadienne Du Vieillissement **26**(2), 159-162. doi:10.3138/cja.26.2.009

Chiari, L., M. Dozza, et al. (2005). "Audio-biofeedback for balance improvement: an accelerometry-based system." IEEE Trans Biomed Eng **52**(12): 2108-2111.

Connelly, D. M., Thomas, B. K., et al. (2009). "Clinical utility of the 2-minute walk test for older adults living in long-term care." Physiotherapy Canada **61**(2): 78-87.

Dozza, M., L. Chiari, et al. (2005). "Influence of a portable audio-biofeedback device on structural properties of postural sway." J Neuroengineering Rehabil **2**: 13.

Dozza, M., L. Chiari, et al. (2005). "Audio-biofeedback improves balance in patients with bilateral vestibular loss." Arch Phys Med Rehabil **86**(7): 1401-1403.

Dozza, M., F. B. Horak, et al. (2007). "Auditory biofeedback substitutes for loss of sensory information in maintaining stance." Exp Brain Res **178**(1): 37-48.

Geurts, A. C., T. W. Mulder, et al. (1992). "Postural organization in patients with hereditary motor and sensory neuropathy." Archives of Physical Medicine and Rehabilitation **73**(6): 569-572.

Hardy, S. E., S. Perera, et al. (2007). "Improvement in usual gait speed predicts better survival in older adults." J Am Geriatr Soc **55**(11): 1727-1734.

Harris, M., R. Eastman, et al. (1993). "Symptoms of sensory neuropathy in adults with NIDDM in the United-States population." Diabetes Care **16**(11): 1446-1452.

Hiengkaew, V., K. Jitaree, et al. (2012). "Minimal detectable changes of the berg balance scale, fugl-meyer assessment scale, timed "up & go" test, gait speeds, and 2-minute walk test in individuals with chronic stroke with different degrees of ankle plantarflexor tone." Arch Phys Med Rehabil **93**(7): 1201-1208.

Horak, F. B., M. Dozza, et al. (2009). "Vibrotactile biofeedback improves tandem gait in patients with unilateral vestibular loss." Ann N Y Acad Sci **1164**: 279-281.

Horak, F. B. (2010). "Postural compensation for vestibular loss and implications for rehabilitation." Restor Neurol Neurosci **28**(1): 57-68.

Inglis, J. T., F. B. Horak, et al. (1994). "The importance of somatosensory information in triggering and scaling automatic postural responses in humans." Experimental Brain Research **101**(1): 159-164.

Leddy, A. L., B. E. Crowner, et al. (2011). "Functional gait assessment and balance evaluation system test: reliability, validity, sensitivity, and specificity for identifying individuals with Parkinson disease who fall." Phys Ther **91**(1): 102-113.

Lin, J. H., M. J. Hsu, et al. (2010). "Psychometric comparisons of 3 functional ambulation measures for patients with stroke." Stroke **41**(9): 2021-2025.

Marchetti, G. F. and S. L. Whitney (2010). Responsiveness and minimal clinically important difference for the Dynamic Gait Index and Functional Gait Assessment in measuring gait performance in patients with balance and vestibular disorders. American Physical Therapy Association Combined Sections Meeting San Diego.

Martyn, C. N. and R. A. Hughes (1997). "Epidemiology of peripheral neuropathy." J Neurol Neurosurg Psychiatry **62**(4): 310-318.

Perera, S., S. H. Mody, et al. (2006). "Meaningful change and responsiveness in common physical performance measures in older adults." J Am Geriatr Soc **54**(5): 743-749.

Powell, L. E. & Myers, A. M. 1995. The Activities-specific Balance Confidence (ABC) Scale. J Gerontol A Biol Sci Med Sci, **50A**(1): M28-M34.

Province, M. A., E. C. Hadley, et al. (1995). "The effects of exercise on falls in elderly patients. A preplanned meta-analysis of the FICSIT Trials. Frailty and Injuries: Cooperative Studies of Intervention Techniques." JAMA **273**(17): 1341-1347.

Richardson, J. K., C. Ching, et al. (1992). "The relationship between electromyographically documented peripheral neuropathy and falls." Journal of the American Geriatrics Society **40**(10): 1008-1012.

Richardson, J. K. and E. A. Hurvitz (1995). "Peripheral neuropathy - a true risk factor for falls." J Gerontol A Biol Sci Med Sci **50**(4): M211-215.

Richardson, J. K. and J. A. Ashton-Miller (1996). "Peripheral neuropathy: an often-overlooked cause of falls in the elderly." Postgrad Med **99**(6): 161-172.

Rubenstein, L. Z., A. S. Robbins, et al. (1990). "The value of assessing falls in an elderly population. A randomized clinical trial." Ann Intern Med **113**(4): 308-316.

Rupert, A. H. (2000). "An instrumentation solution for reducing spatial disorientation mishaps." IEEE Eng Med Biol Mag **19**(2): 71-80.

Rupert, A. H. (2000). "Tactile situation awareness system: proprioceptive prostheses for sensory deficiencies." Aviat Space Environ Med **71**(9 Suppl): A92-99.

Studenski, S., P. W. Duncan, et al. (1991). "Postural responses and effector factors in persons with unexplained falls: results and methodologic issues." J Am Geriatr Soc **39**(3): 229-234.

Statler, K., D. Wrisley, et al. (2007). Vibrotactile feedback of medial-lateral trunk tilt or foot pressure reduces risk of falling in healthy older adults. International Society for Posture & Gait, 18th International Conference, Burlington, VT, ISPG.

Tyler, M., Y. Danilov, et al. (2003). "Closing an open-loop control system: vestibular substitution through the tongue." J Integr Neurosci **2**(2): 159-164.

Wall, C., 3rd (2010). "Application of vibrotactile feedback of body motion to improve rehabilitation in individuals with imbalance." J Neurol Phys Ther **34**(2): 98-104.

Wall, C., 3rd and E. Kentala (2010). "Effect of displacement, velocity, and combined vibrotactile tilt feedback on postural control of vestibulopathic subjects." J Vestib Res **20**(1): 61-69.

Wall, C., 3rd, D. M. Merfeld, et al. (2002). "Vestibular prostheses: the engineering and biomedical issues." J Vestib Res **12**(2-3): 95-113.

Wall, C., 3rd, L. E. Oddsson, et al. (2004). "Applications of vibrotactile display of body tilt for rehabilitation."Conf Proc IEEE Eng Med Biol Soc **7**: 4763-4765.

Wall, C., 3rd and M. S. Weinberg (2003). "Balance prostheses for postural control." IEEE Eng Med Biol Mag **22**(2): 84-90.

Wall, C., 3rd, M. S. Weinberg, et al. (2001). "Balance prosthesis based on micromechanical sensors using vibrotactile feedback of tilt." IEEE Trans Biomed Eng **48**(10): 1153-1161.

Wall, C., 3rd, D. M. Wrisley, et al. (2009). "Vibrotactile tilt feedback improves dynamic gait index: a fall risk indicator in older adults." Gait Posture **30**(1): 16-21.

Wall, C. W., D.; Oddsson, L. (2012). Vibrotactile Feedback of Mediolateral Trunk Tilt or Foot Pressure Increases Locomotor Performance in Healthy Older Adults - a Pilot Study. 34th Annual International Conference of the IEEE Engineering in Medicine and Biology Society (EMBC). San Diego, Conf Proc IEEE Eng Med Biol Soc.

Wrisley, D. M. and N. A. Kumar (2010). "Functional gait assessment: concurrent, discriminative, and predictive validity in community-dwelling older adults." Phys Ther **90**(5): 761-773.

Wrisley, D. M., G. F. Marchetti, et al. (2004). "Reliability, Internal Consistency, and Validity of Data Obtained With the Functional Gait Assessment." PHYS THER **84**(10): 906-918.

**Appendix: Activities-specific Balance Confidence Scale**

**
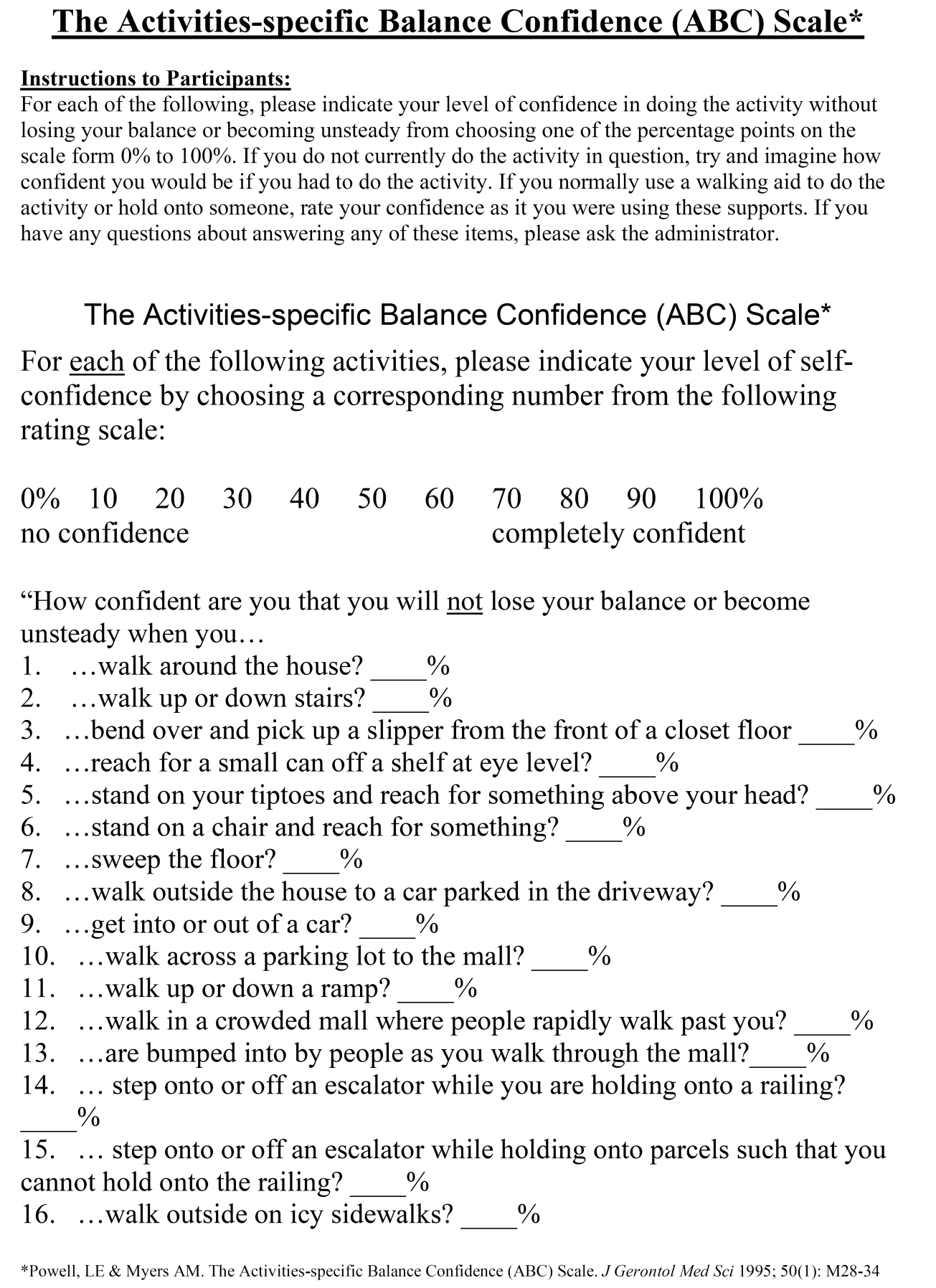
**
